# 

**Published:** 1921-03

**Authors:** 


### ANNOUNCEMENTS

---

### American Institute of Dental Teachers

At the annual meeting of the American Institute of Dental Teachers, held at Indianapolis, Ind., Jan. 24, 25 and 26, the following officers were elected for the ensuing year: President, Dr. Guy S. Millberry, University of California, San Francisco, Cal.; Vice-President, Dr. A. H. Hipple, Creighton University, Omaha, Neb.; Secretary-Treasurer, Dr. Abram Hoffman, 381 Linwood Ave., Buffalo, N. Y.; Executive Board, Dr. A. E. Webster, Royal College of Dental Surgeons, Toronto, Ont., one year; Dr. E. D. Coolidge, University of Illinois, Chicago, Ill., two years; Dr. H. E. Wheeler, College of Dental and Oral Surgery, New York City, three years.

The next meeting will be held the third week in January, 1922, at Montreal, Que.

